# Enhanced Tetracycline Adsorption Using KOH-Modified Biochar Derived from Waste Activated Sludge in Aqueous Solutions

**DOI:** 10.3390/toxics12100691

**Published:** 2024-09-25

**Authors:** Jiazheng Ding, Jiahao Liang, Qinghong Wang, Xiang Tan, Wenyu Xie, Chunmao Chen, Changgang Li, Dehao Li, Jin Li, Xiaoqing Chen

**Affiliations:** 1 Key Laboratory of Petrochemical Pollution Control of Guangdong Higher Education Institutes, Guangdong Engineering Technology Research Center of Petrochemical Pollution Control, School of Environmental Science and Engineering, Guangdong University of Petrochemical Technology, Maoming 525000, China; dingjz1999@163.com (J.D.); gdtanxiang@163.com (X.T.); gdmmxwy@gdupt.edu.cn (W.X.); dehlee@163.com (D.L.); 2 State Key Laboratory of Heavy Oil Processing, State Key Laboratory of Petroleum Pollution Control, China University of Petroleum-Beijing, Beijing 102249, China; wangqhqh@163.com (Q.W.); c.chen@cup.edu.cn (C.C.); lijincup@163.com (J.L.); 3 School of Energy and Power Engineering, Guangdong University of Petrochemical Technology, Maoming 525000, China; chenxiaoqing0710@163.com; 4 Institute of Science and Environment, University of Saint Joseph, Macau 999078, China

**Keywords:** sludge biochar, tetracycline, adsorption, waste activated sludge, KOH modification

## Abstract

Antibiotic pollution poses a serious environmental concern worldwide, posing risks to ecosystems and human well-being. Transforming waste activated sludge into adsorbents for antibiotic removal aligns with the concept of utilizing waste to treat waste. However, the adsorption efficiency of these adsorbents is currently limited. This study identified KOH modification as the most effective method for enhancing tetracycline (TC) adsorption by sludge biochar through a comparative analysis of acid, alkali, and oxidant modifications. The adsorption characteristics of TC upon unmodified sludge biochar (BC) as well as KOH-modified sludge biochar (BC-KOH) were investigated in terms of equilibrium, kinetics, and thermodynamics. BC-KOH exhibited higher porosity, greater specific surface area, and increased abundance of oxygen-based functional groups compared to BC. The TC adsorption on BC-KOH conformed the Elovich and Langmuir models, with a maximum adsorption capacity of 243.3 mg/g at 298 K. The adsorption mechanisms included ion exchange, hydrogen bonding, pore filling, and electrostatic adsorption, as well as π-π interactions. Interference with TC adsorption on BC-KOH was observed with HCO_3_^−^, PO_4_^3−^, Ca^2+^, and Mg^2+^, whereas Cl^−^, NO_3_^−^, and SO_4_^2−^ ions exhibited minimal impact on the adsorption process. Following three cycles of utilization, there was a slight 5.94% reduction in the equilibrium adsorption capacity, yet the adsorption capacity remained 4.5 times greater than that of unmodified sludge BC, underscoring its significant potential for practical applications. This research provided new insights to the production and application of sludge biochar for treating antibiotic-contaminated wastewater.

## 1. Introduction

Tetracycline (TC), a prevalent antibiotic, is frequently prescribed for bacterial, rickettsial, chlamydial, and mycoplasmal infections [1]. Due to its chemical durability and notable resistance to biodegradation, an estimated 70–90% of TC remains unmetabolized in both human and animal systems, eventually entering wastewater treatment facilities or the environment via excretion in feces and urine [2]. TC is recognized for its limited biodegradability and is commonly observed in wastewater treatment plant effluents, varying from 0 to 32,000 µg/L [3]. Given the ecological and environmental hazards linked to antibiotic contamination, it is imperative to advance sustainable technologies for eliminating TC from wastewater [4,5]. However, conventional processes (e.g., biological treatment, filtration, coagulation, and sedimentation) are unlikely to eliminate trace antibiotics from wastewater [6].

Adsorption represents an environmentally friendly, cost-efficient, and highly effective wastewater treatment technology that can be implemented without generating harmful byproducts [7]. Notably, the adsorbents utilized for pollutant capture can be centrally processed, mitigating potential environmental hazards. Activated carbon is commonly employed in wastewater treatment due to its cost-effectiveness, wide accessibility, and superior adsorption capabilities. Sewage sludge, a byproduct from the biological processes of wastewater treatment facilities, is a waste material. Globally, an annual production of 80 to 90 million tons of waste activated sludge is observed, with a consistent upward trend, presenting a significant challenge to both the social economy and environment [8,9]. As a carbonaceous material, the waste activated sludge can be transformed into a biochar adsorbent through pyrolysis, addressing solid waste issues and harnessing sludge resources [10]. Nevertheless, because of inadequate pore structure and active sites within biochar produced directly through pyrolysis, its adsorption capacity for TC often falls below 40 mg/g [11,12,13,14]. Chemical activation is a proven and effective method to enhance the adsorption capacity of biochar [15,16]. Yu et al. demonstrated the following KOH activation, in which the specific surface area of sludge biochar rose from 30.39 to 44.71 m^2^/g, and the maximum TC adsorption capacity improved from 25 to 50.75 mg/g [17]. Additionally, chemical modifications have also been documented to elevate the specific surface area and porosity, as well as active sites upon biochar, thereby boosting its adsorption capacity [18]. It is noteworthy that, unlike chemical activation, chemical modification can be conducted at room temperature and does not require an oxygen-deficient environment, making it more feasible for industrial applications. Acid-modified biochar typically exhibited increased pore volume and a broader spectrum of pore sizes [19]. The modifications with oxidants were shown to increase the abundance of oxygen-containing functional groups and the quantity of micropores in biochar [20], while alkali treatments significantly increased the specific surface area and enhanced π-π interactions with aromatic rings on the surface [21,22]. Thus, chemical modifications are expected to enhance TC adsorption onto sludge biochar. However, related studies are still scarce.

This research found that KOH treatment was the most effective method when comparing different acid, alkali, and oxidant treatments for sludge biochar in the adsorption capacity of TC. This work systematically studied how KOH modification has affected the properties of sludge biochar. It assessed the TC adsorption capabilities of sludge biochar modified with KOH, as well as its resistance to ion interference and reusability. Furthermore, the study investigated the adsorption kinetics, thermodynamics, and mechanisms involved. The research provides innovative perspectives into the creation and utilization of sludge biochar to treat antibiotic-containing wastewater.

## 2. Materials and Methods

### 2.1. Waste Activated Sludge

Waste activated sludge was supplied by a sludge dewatering workshop at the Hexi Wastewater Treatment Plant in Maoming city, China. It exhibited a moisture content of 78% and a volatile suspended solids (VSS) to total suspended solids (TSS) ratio of 0.5. The sludge was first oven-dried at 60 °C for 24 h before being sieved to achieve a granularity of less than 200 mesh.

### 2.2. Preparation of Sludge Biochar and Chemical Modification

Biochar from sludge was produced via pyrolysis in a tubular furnace (KJ-T1700-L8010LB, Zhengzhou, China). The sludge was gradually heated at a rate of 5 °C/min under an argon flow of 100 mL/min until it reached a temperature of 600 °C. This process took 2 h. The biochar was washed multiple times with ultrapure water and then labeled as BC. Next, 10 g of the dried BC was placed in a solution containing various modifiers (KOH, NaOH, NaHCO_3_, K_3_PO_4_, H_3_PO_4_, H_2_SO_4_, HNO_3_, KMnO_4_, H_2_O_2_) with a concentration of 2 mol/L. The mixture was stirred using a magnetic stirrer at a temperature of 25 °C for 12 h and then dried in an oven at 105 °C for another 12 h. These biochars underwent a process of acid-washing using 5% HCl and were rinsed multiple times with deionized water until they reached a neutral pH. Afterward, the biochars underwent a drying process at 60 °C and were then carefully sifted to ensure a particle size smaller than 120 mesh. The preparation steps are detailed in Figure S1. For clarity in result descriptions, the biochars modified using KOH, NaOH, NaHCO_3_, K_3_PO_4_, H_3_PO_4_, H_2_SO_4_, HNO_3_, KMnO_4_, and H_2_O_2_ were labeled as BC-KOH, BC-NaOH, BC-NaHCO_3_, BC-K_3_PO_4_, BC-H_3_PO_4_, BC-H_2_SO_4_, BC-HNO_3_, BC-KMnO_4_, and BC-H_2_O_2_, respectively.

### 2.3. Batch Adsorption Experiments

#### 2.3.1. Evaluation of TC Adsorption Performance of Different Sludge Biochars

The adsorptive performance of unmodified and modified sludge biochar (BC, BC-KOH, BC-NaOH, BC-NaHCO_3_, BC-K_3_PO_4_, BC-H_3_PO_4_, BC-H_2_SO_4_, BC-HNO_3_, BC-KMnO_4_, and BC-H_2_O_2_) for TC was evaluated on a batch basis. A total of 100 mg of TC was dissolved in 1 L of deionized water. The solution containing 100 mg/L of TC was adjusted to pH 7.0 with 0.1 mol/L HCl or NaOH, as most TC-containing wastewater was neutral [23]. In all the experiments, 25 mg sludge biochar was mixed with 50 mL of the TC solution in a conical flask and agitated at 180 rpm at 25 °C. After 24 h, the samples were filtered, and the TC concentrations measured at 356 nm [13].

#### 2.3.2. Effect of Coexisting Ions on TC Adsorption

To assess the influence of various ions (Cl^−^, NO_3_^−^, HCO_3_^−^, SO_4_^2−^, PO_4_^3−^, Ca^2+^, and Mg^2+^) and their concentrations on TC adsorption, NaCl, NaNO_3_, NaHCO_3_, Na_2_SO_4_, Na_3_PO_4_, CaCl_2_, and MgCl_2_ were introduced into a 100 mg/L TC solution at concentrations of 0.05 or 0.1 mol/L. Using a similar approach, 25 mg of sludge biochar was introduced into 50 mL of the TC solution and stirred for 24 h. After passing through a 0.45 μm membrane, the concentration of TC was determined.

#### 2.3.3. Effect of pH on TC Adsorption

The pH of the 100 mg/L TC solution was adjusted to a range of 2 to 12 using either 0.1 mol/L NaOH or 0.1 mol/L HCl. Subsequently, 50 mL of the pH-adjusted TC solution was combined with 25 mg of sludge biochar in a 100 mL conical flask, agitated at 180 rpm and 25 °C for 24 h. After that, the TC concentration was measured following filtration.

#### 2.3.4. Reusability Evaluation

A conical flask containing TC solution was agitated under the same conditions. Following the adsorption period, the biochar with adsorbed TC was collected and treated with a 50% ethanol solution. It was then subjected to a 2 h ultrasonic treatment to aid in TC desorption. Subsequently, the biochar was dried at 60 °C, milled, and passed through a 120-mesh sieve to achieve a particle size. This regenerated sludge biochar was subsequently used in the next phase of adsorption experiments.

#### 2.3.5. Adsorption Kinetics, Isotherm and Thermodynamics

Adsorption kinetics tests were conducted with 50 mL TC solution at pH 7. Following this, 25 mg of sludge biochar was introduced, and then the mixture was agitated at 298 K and 180 rpm. Samples were collected at 10, 20, 30, 60, 90, 120, 180, 240, 360, 540, 720, 1080, and 1440 min. After filtration, the TC concentration in each sample was determined and the resulting adsorption curve was analyzed using pseudo-first-order, pseudo-second-order, Elovich, intra-particle diffusion, and liquid film diffusion models [24,25]. The detailed equations used for data fitting are provided in the Supplementary Materials.

For the adsorption isotherm studies, 25 mg of sludge biochar was added to 50 mL of TC solution at pH 7, with varying concentrations of 10, 20, 40, 60, 80, 100, 120, and 150 mg/L. The adsorption experiments were carried out for a duration of 24 h at three different temperatures: 288 K, 298 K, and 308 K. Throughout the experiments, there was constant agitation at a rate of 180 rpm. After adsorption, the concentration of TC was measured. The adsorption isotherm data were then fitted using the Langmuir, Freundlich, and Temkin models [13,26]. These detailed equations used for data fitting are provided in the Supplementary Materials.

### 2.4. Analytical Methods

The specific surface area as well as porous characteristics of sludge biochar were examined through an ASAP2020HD analyzer. The surface morphology was examined using a scanning electron microscopy (SEM) (Gemini 300, ZEISS Ltd., Aalen, Germany). Surface functional groups were identified using a Fourier transform infrared (FTIR) spectrometer (Shimadzu IRAffinity-1S, Shimadzu Corp, Tokyo, Japan). Surface elemental composition and state were determined by a X-ray photoelectron spectroscopy (XPS) (Escalab Xi+, Thermo Fisher Inc., Shanghai, China). Graphitization was examined using a Raman spectrometer (Renishaw 2000, Renishaw Ltd, Gloucestershire, UK) at 532 nm. The point of zero charge (pH_PZC_) was determined through a solid addition method [27].

## 3. Results and Discussions

### 3.1. The Adsorption Capacity Comparison of Different Modified Sludge Biochars towards TC

**Figure 1:**
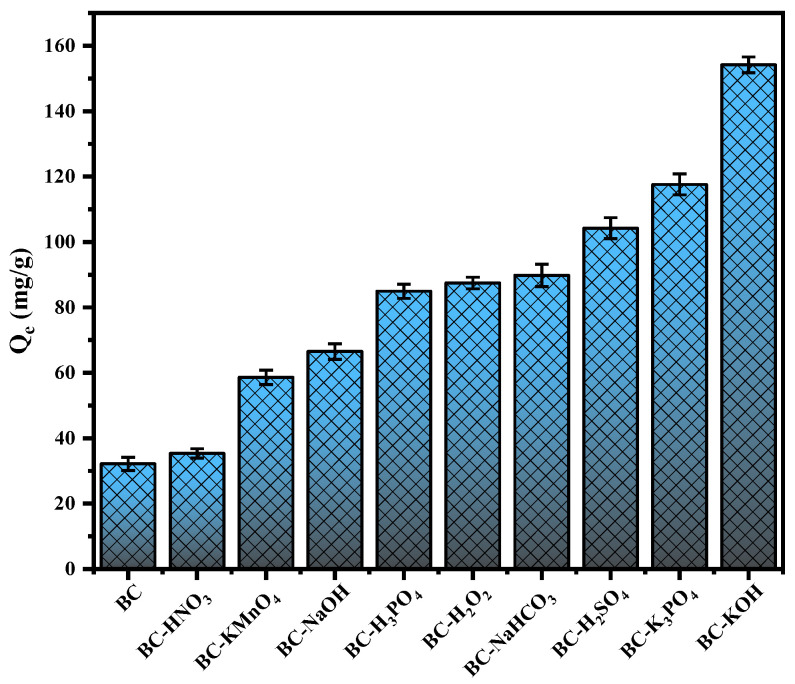
TC adsorption capacity of different modified biochar.

Figure 1 illustrates the TC adsorption capacity under various modified biochars. As observed in previous studies [11,12,13,14], the TC adsorption capacity of sludge biochar consistently remained below 40 mg/g, with an equilibrium adsorption quantity of merely 30.6 mg/g. However, its capacity for equilibrium adsorption exhibited varying degrees of enhancement through modification with alkalis (KOH, NaOH, NaHCO_3_, K_3_PO_4_), acids (H_3_PO_4_, H_2_SO_4_, HNO_3_), and oxidants (KMnO_4_, H_2_O_2_). Among them, KOH modification exhibited the greatest increase in TC adsorption for sludge biochar. The equilibrium adsorption amount of BC-KOH reached 154.16 mg/g, representing a 4.8-fold increase compared to the unmodified BC. Surprisingly, the adsorption capacity of BC-KOH was three times higher than that of KOH-activated sludge biochar, as previously published by Yu et al. [17]. Therefore, this study was to investigate how KOH modifications affect TC adsorption by sludge biochar.

### 3.2. Characterization of BC and BC-KOH

#### 3.2.1. Morphology and Porosity Structure of Sludge Biochar

**Figure 2:**
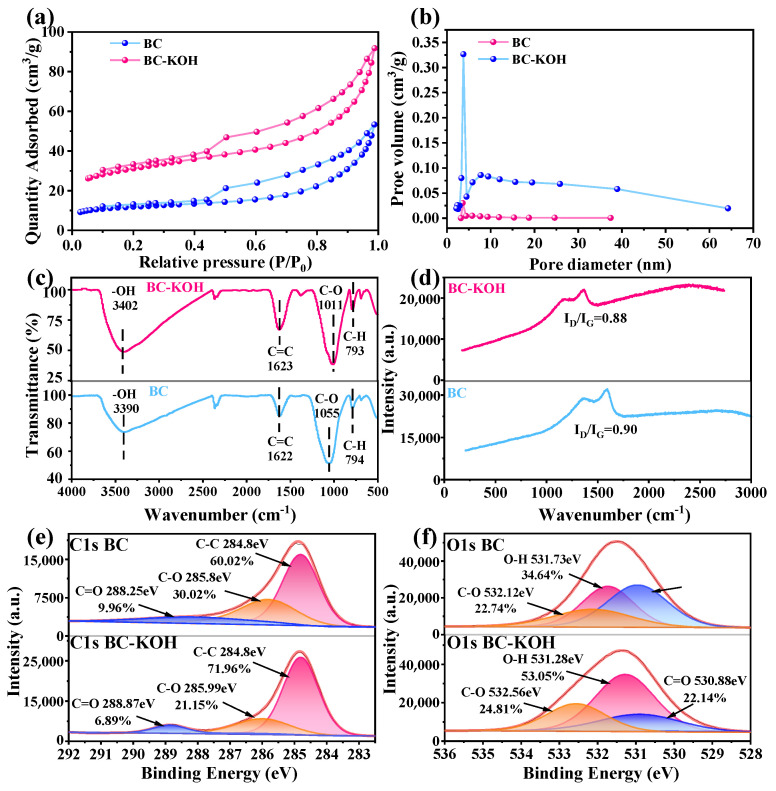
(**a**) Nitrogen adsorption–desorption isotherm curves of BC and BC-KOH; (**b**) pore-size distribution curves of BC and BC-KOH; (**c**) FTIR spectra of BC and BC-KOH; (**d**) Raman spectra of BC and BC-KOH; (**e**) XPS of C1s for BC and BC-KOH; (**f**) XPS of O1s for BC and BC-KOH.

SEM images (Figure S2) revealed that both BC and BC-KOH exhibited irregular pores and rough surfaces. A higher density of pores was evident in BC-KOH, indicating that KOH modification promoted pore development. The abundant porous structure could provide more active sites and enhance pore diffusion efficiency for TC adsorption [28]. Brunauer–Emmett–Teller (BET) measurements further confirmed the impact of KOH modification in promoting pore formation. BC-KOH exhibited a 72.1% increase in total pore volume and a 197.1% increase in microporous volume compared to BC (Table 1). The specific surface area demonstrated a marked increase from 43.7 m^2^/g in BC to 112.5 m^2^/g in BC-KOH. Both samples exhibited a distinct Type IV nitrogen adsorption–desorption curve, featuring an H4-type hysteresis loop (Figure 2a). This indicates that the pore structures of BC and BC-KOH are predominantly mesoporous (2–50 nm) and microporous (<2 nm) [29], with BC-KOH showing more of them (Figure 2b). Especially, the number of mesopores range of 5–50 nm in BC-KOH was significantly greater than that in BC. The result illustrated that KOH modification significantly enhanced the porosity of the sludge biochar, critical factors for improved adsorption performance [30].

#### 3.2.2. Surface Functional Groups and Graphitization of Sludge Biochar

In Figure 2c, the infrared spectra of BC and BC-KOH are shown. The peaks at around 1622 and 3390 cm^−1^ represent the stretching vibrations of the C=C bond and the O-H bond in the benzene ring, respectively [31,32,33]. The peaks at 1011 and 1055 cm^−1^ were the result of stretching vibrations in C-O [34]. These peaks showed that hydroxyl groups and benzene rings existed on the surfaces of both BC and BC-KOH. However, after modification by KOH, more hydroxyl and benzene ring groups were formed, as evidenced by the increase in the intensity of the mentioned peaks in BC-KOH.

Figure 2d illustrates the Raman spectra of BC and BC-KOH. The peak at 1360–1380 cm^−1^ corresponded to a D peak, generated by sp^3^ hybrid carbon atoms at defect positions in the biochar. Additionally, the peak between 1570 and 1590 cm^−1^ corresponded to the G peak produced by the vibration of sp^2^ hybrid carbon atoms within the biochar [35]. The positions of D (1368 cm^−1^) and G (1580 cm^−1^) peaks within BC differed from the D (1356 cm^−1^) and G (1585 cm^−1^) peaks within BC-KOH. The I_D_/I_G_ ratio of BC was 0.90, while of BC-KOH was 0.88, suggesting a higher degree of graphitization in BC-KOH [36].

The C1s spectra of BC (Figure 2e) suggested an existence of C-C (284.80 eV), C-O (285.8 eV), and C=O (288.25 eV) functional groups, with a relative content of 60.02%, 30.02%, and 9.96%, respectively. Upon KOH modification, BC-KOH exhibited a higher degree of graphitization, with a relative content of C-C groups rising from 60.02% up to 71.96%. This observation suggested that KOH modification altered the skeleton structure of the sludge biochar, causing a shift within the composition of functional groups. This O1s spectra of BC (Figure 2f) displayed three peaks at 530.95 eV (C=O), 531.73 eV (O-H), as well as at 532.12 eV (C-O) [37]. After KOH modification, the percentage of O-H content increased from 34.64% to 53.05%, while the C-O content rose from 22.74% up to 24.81%. These findings suggested the generation of hydroxyl groups on the sludge biochar’s surface following modification with KOH, consistent with the FTIR spectral analysis. This phenomenon was likely attributed to the chemical interaction between the free radicals released by KOH and the carbon and oxygen atoms in the biochar during the modification.

### 3.3. Adsorption Kinetics, Isotherms, Thermodynamics, and Influence Factor

#### 3.3.1. Adsorption Kinetics

**Figure 3:**
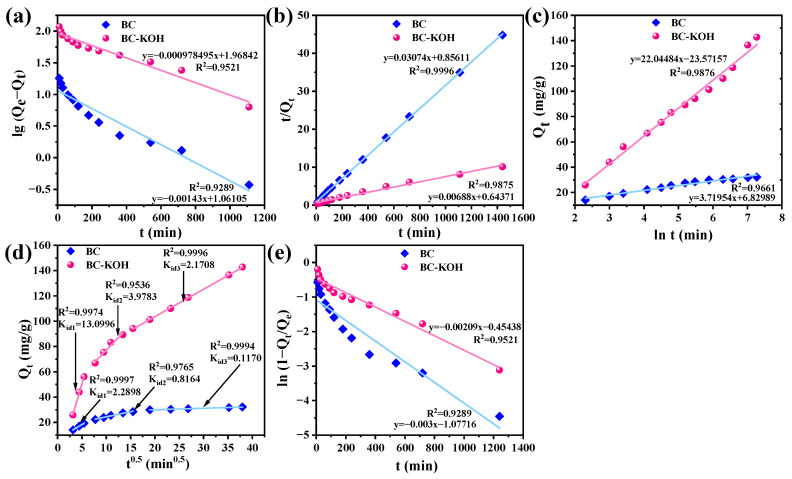
Effect of contact time on the TC adsorption by BC, BC-KOH: (**a**) the pseudo-first-order model; (**b**) the pseudo-second-order model; (**c**) the Elovich model; (**d**) the intraparticle model; (**e**) liquid membrane diffusion models.

The adsorption kinetics of TC by sludge biochar were assessed utilizing pseudo-first-order, pseudo-second-order, and Elovich models. Data are detailed in Figure 3a–c and Table S1. The correlation coefficient (R^2^) for the pseudo-second-order model in BC was 0.9996, exceeding those of the pseudo-first-order model (0.9289) and the Elovich model (0.9661), indicating that the pseudo-second-order model more accurately described the adsorption kinetics of TC by BC. This suggested that chemical adsorption was the predominant mechanism in the adsorption process [38]. For BC-KOH, the Elovich model displayed the highest R^2^ at 0.9876, surpassing both the pseudo-first-order model (0.9521) and the pseudo-second-order model (0.9875), demonstrating that TC adsorption by BC-KOH aligned more accurately with the Elovich model. This suggested an uneven energy distribution upon the surface of BC-KOH, with chemical adsorption being a prevalent mechanism during TC adsorption [39,40].

Such adsorption kinetics were further fitted using the intraparticle diffusion model (Figure 3d) and the liquid film diffusion model (Figure 3e), respectively, to elucidate the diffusion mechanism involved. Intraparticle diffusion plots for both BC and BC-KOH were multilinear, which indicated the three-stage process of TC adsorption involving external diffusion, internal diffusion within particles, and adsorption at the active sites on the sludge biochar. The high determination coefficients R^2^ exceeding 0.95 for BC and BC-KOH in the intraparticle diffusion model suggested that intraparticle diffusion was the rate-limiting step for TC adsorption. These intraparticle diffusion rate constant (K_id_) values for BC-KOH were 3.9 to 17.6 times higher than that for BC across the three adsorption stages. This enhancement might be ascribed to the improved pore structure of biochar resulting from KOH modification. Moreover, the liquid film diffusion model also fitted well for both BC and BC-KOH, with R² values exceeding 0.90. This indicated a significant role for liquid film diffusion in the rate-limiting stage of TC adsorption, consistent with the findings of Chang et al. [41].

#### 3.3.2. Adsorption Isotherms

**Figure 4:**
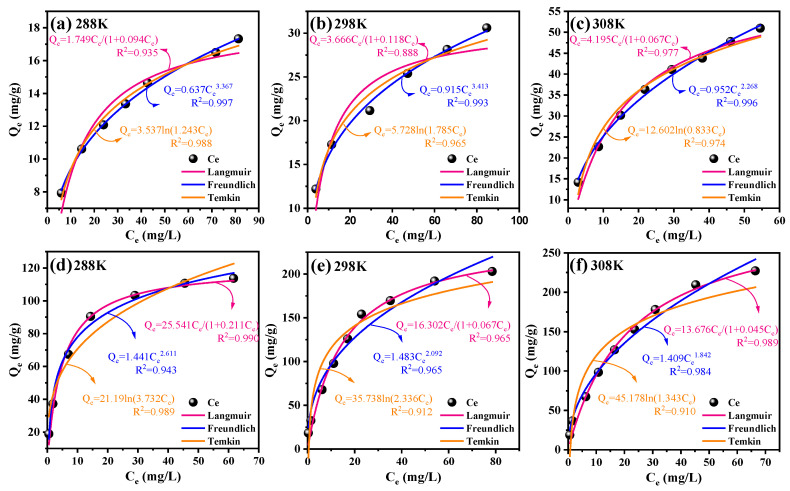
Adsorption isotherms of TC on BC, BC-KOH: (**a**) BC 288K; (**b**) BC 298K; (**c**) BC 308K; (**d**) BC-KOH 288K; (**e**) BC-KOH 298K; (**f**) BC-KOH 308K.

Figure 4 illustrates the adsorption isotherms at various temperatures. The adsorption behavior of BC correlated well with the Freundlich isotherm model, achieving R^2^ values of 0.997 at 288 K, 0.993 at 298 K, and 0.996 at 308 K. The findings suggested that TC adsorption through BC involved multilayer adsorption with varying binding sites [42]. TC molecules were uniformly adsorbed onto a monolayer of adsorption sites on BC-KOH, as TC adsorption with this sorbent showed better compliance with the Langmuir isotherm model than the Freundlich and Temkin models (Table S2) [43]. Furthermore, it was noted that the adsorption capabilities of both BC and BC-KOH increased as the temperature rose, suggesting the occurrence of an endothermic reaction. At 288 K, the maximum adsorption capacity of BC-KOH was 121.05 mg/g. At 298 K, it increased to 243.32 mg/g, and at 308 K, it further increased to 303.90 mg/g. These values represented a 5.5-fold, 6.8-fold, and 3.9-fold increase, respectively, compared to the adsorption capacities of BC under the same conditions. When compared to other biomass-derived carbon materials (Table 2), the maximum adsorption capacity of BC-KOH exceeded that of most biochar materials, highlighting its significant potential for removing TC antibiotics from wastewater.

#### 3.3.3. Adsorption Thermodynamics

This research analyzed the thermodynamic properties of TC adsorption on sludge biochar. Table 3 displays various thermodynamic parameters, such as Gibbs free energy (Δ*G*^0^), enthalpy (Δ*H*^0^), and entropy (Δ*S*^0^). The positive Δ*G*^0^ value for BC adsorption implied that it was not spontaneous. Conversely, the negative Δ*G*^0^ for BC-KOH adsorption suggested it was spontaneous and thermodynamically favorable. Furthermore, both BC and BC-KOH exhibited a reduction in Δ*G*^0^ as the adsorption temperature increased, indicating higher temperatures favor the adsorption process, as evidenced by the increase in equilibrium adsorption capacity. With rising temperatures, the solid–liquid adsorption system gained more degrees of freedom, leading to an increased frequency of contact among the active sites on the biochar surface with TC molecules, thereby boosting the adsorption capacity [49]. Both BC and BC-KOH recorded positive Δ*H*^0^ values, signifying that the adsorption process for TC was endothermic, with elevated temperatures facilitating increased adsorption. Notably, BC-KOH exhibited higher Δ*H*^0^ and Δ*S*^0^ values, indicating a more pronounced affinity for TC, thereby facilitating the adsorption process [50,51].

#### 3.3.4. The Effect of pH on the Adsorption of TC

**Figure 5:**
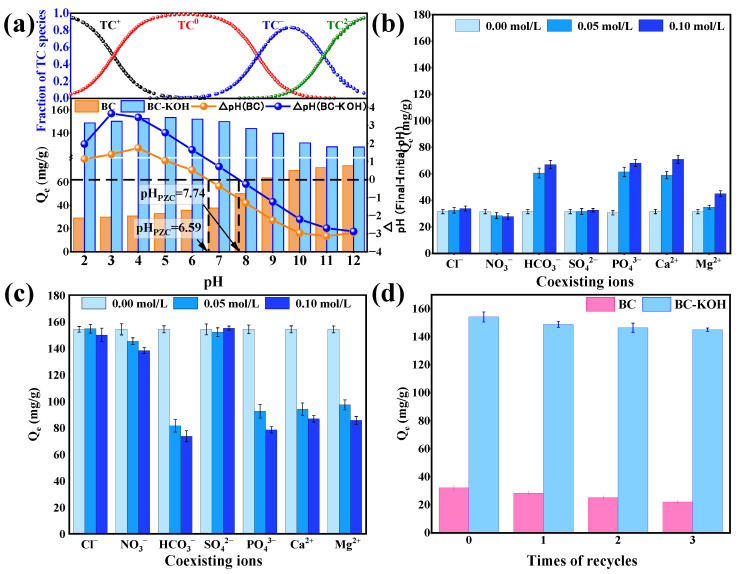
(**a**) Influence of pH on adsorption; (**b**) effect of coexisting anions on the adsorption of TC by BC; (**c**) effect of coexisting anions on the adsorption of TC by BC-KOH; (**d**) effect of cycle times on TC removal.

The solution pH can significantly impact the surface charge of the sludge biochar as well as the ionization state of TC, thereby influencing its adsorption capacity [52]. The pH_PZC_ for BC and BC-KOH were determined to be 6.59 and 7.74, respectively (Figure 5a). The higher pH_PZC_ of BC-KOH was likely because of the interaction among KOH with acidic functional groups on the biochar surface during modification [53]. These adsorption characteristics of BC and BC-KOH exhibited distinct trends with changing pH. The BC adsorption capacity elevated as the pH rose. Below the pH_PZC_ (6.59), the predominant TC species were TC^+^ and TC^0^. As pH increased, the proportion of TC^+^ decreased, consequently decreasing the electrostatic repulsion among positively charged BC with TC. Upon surpassing pH_PZC_, the fraction of TC^−^/TC^2−^ increased, leading to increased electrostatic repulsion among these negatively charged species and the BC surface. However, the increased pH also altered the electron distribution within the aromatic rings of TC and improved the pore structure of BC, thereby enhancing π-π interactions as well as the pore-filling capacity between TC and BC. For BC-KOH, the adsorption capacity initially showed an increase and then a decrease as pH rose. Within the pH range of 2 and 5, the reduction within proportion of TC^+^ species diminished the electrostatic repulsion among positively charged BC-KOH and TC, resulting in increased adsorption. Beyond pH 5, the proportion of TC^−^ weakened the hydrogen bond interactions between TC^−^ and the carbon–oxygen groups that were negatively charged on the BC-KOH surface, thereby slightly diminishing the adsorption capacity. Upon surpassing the pH_PZC_ of BC-KOH (7.74), TC was primarily presented as TC^−^ and TC^2−^, and the resulting electrostatic repulsion among these negatively charged species with BC-KOH led to a further decrease in adsorption capacity. These observations suggested that electrostatic interactions significantly influence the adsorption of both BC and BC-KOH at different pH levels.

#### 3.3.5. Effect of Coexisting Ions

The existence of both anionic and cationic ions could potentially compete with TC during the adsorption process in real wastewater samples. TC adsorption by both BC and BC-KOH was largely unaffected by Cl^−^, NO_3_^−^, and SO_4_^2−^. However, with increasing concentrations of HCO_3_^−^, PO_4_^3−^, Ca^2+^, and Mg^2+^, TC adsorption by BC showed a continuous upward trend, whereas the adsorption capacity of BC-KOH decreased (Figure 5b,c). Furthermore, the introduction of HCO_3_^−^ and PO_4_^3−^ resulted in an increase in the pH in the TC solution, thereby augmenting TC adsorption by BC while marginally decreasing TC adsorption by BC-KOH. The increased TC adsorption by BC with Ca^2+^ and Mg^2+^ was likely attributed to the neutralization of BC’s negative surface charge, reducing electrostatic repulsion among negatively charged BC as well as TC^−^. In contrast, these larger pore sizes of BC-KOH allowed Ca^2+^ and Mg^2+^ ions into its internal structure, enabling them to occupy active adsorption sites. This resulted in competition with TC for limited binding sites, thereby reducing the adsorption capacity of BC-KOH [16].

#### 3.3.6. Recycle Performance

Figure 5d shows the reusability of BC and BC-KOH treated with 50% ethanol and ultrasound. Following regeneration, both sludge biochars showed a slight reduction in TC adsorption capacities. After three adsorption–desorption cycles, BC’s adsorption capacity diminished from 32.13 mg/g to 22.11 mg/g, a 31.19% reduction, whereas BC-KOH showed a decrease from 154.16 mg/g to 145.01 mg/g, a decline of only 5.94%. This demonstrated that KOH modification significantly enhanced the performance of sludge biochar as an adsorbent and substantially improved its adsorption efficiency, stability, and reusability over multiple cycles. It is important to note that ultrasound treatment can partially degrade TC; however, complete mineralization of TC was not achieved. To mitigate potential environmental risks associated with residual TC in the desorbed solution, additional treatment steps may be required to ensure complete mineralization or degradation. To this end, advanced oxidation processes (AOPs) such as photocatalysis or ozone oxidation could be considered to prevent the reintroduction of TC into the environment.

### 3.4. Adsorption Mechanism

**Figure 6:**
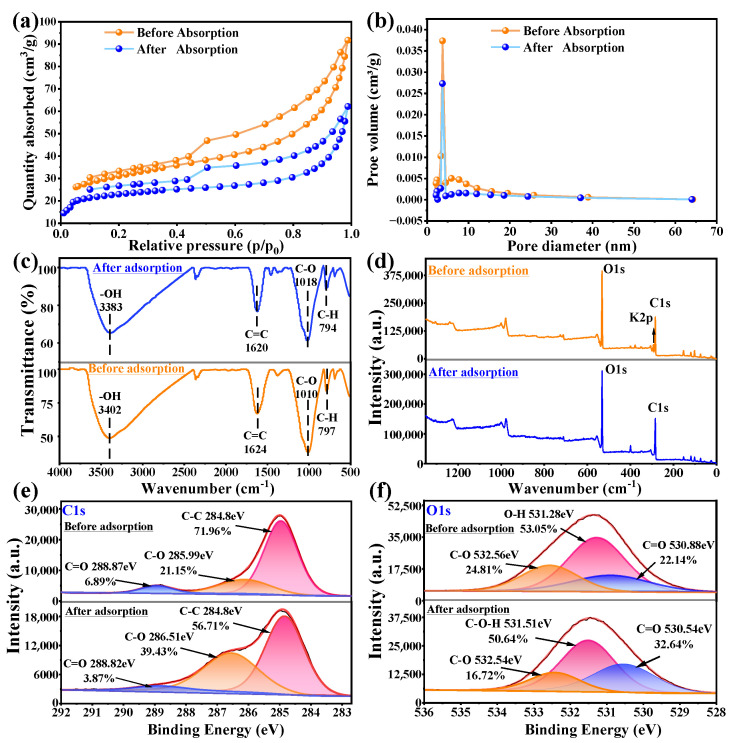
(**a**) The variation of N_2_ adsorption–desorption isotherms of BC-KOH before and after TC removal; (**b**) pore size distribution curves of BC-KOH before and after TC removal; (**c**) FT-IR spectra of BC-KOH before and after TC removal; (**d**) XPS survey of BC-KOH before and after TC removal; (**e**) C1s spectra of XPS of BC-KOH before and after TC removal; (**f**) O1s spectra of XPS of BC-KOH before and after TC removal.

**Figure 7:**
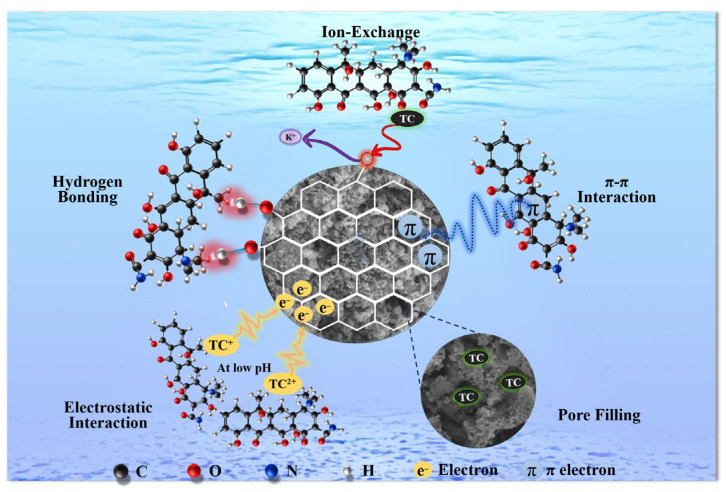
The possible adsorption mechanisms of BC-KOH for TC, reproduced from [60], with permission from Elsevier, 2024.

Analyzing the surface functional groups and pore structure both before and after adsorption was part of the inquiry into the TC adsorption mechanism by BC-KOH. After adsorption, the overall pore volume of BC-KOH decreased from 1.14191 to 0.09612 cm^3^/g, whereas the micropore volume decreased slightly from 0.031 to 0.0306 cm^3^/g (Table S3). These findings suggested that pore filling significantly contributed to the adsorption process [37]. FTIR spectra (Figure 6c) showed shifts in the stretching vibration peaks of -OH and C-O, shifting from 3402 to 3383 cm⁻^1^ and from 1010 cm^−1^ to 1018 cm^−1^, respectively, along with a notable reduction. These changes suggested these oxygen-containing functional groups upon BC-KOH may interact with C=O and -OH groups on TC molecules through hydrogen bonding [54,55,56]. Additionally, the stretching vibration peaks of C=C at 1624 cm^−1^ and C-H at 797 cm^−1^ also shifted and decreased in intensity, possibly because of π-π interactions among aromatic rings on BC-KOH with benzene rings, carboxyl groups, and acid-base groups on TC. These interactions could lead to a weakening of C=C bonds on benzene rings, leading to a wavelength shift in the absorption peaks [57,58]. The XPS spectra of BC-KOH post TC adsorption (Figure 6d) showed the absence of the K2p peak, suggesting the presence of ion exchange among TC with K^+^. The C1s spectra (Figure 6e) showed a reduction in the C-C peak proportion from 71.96% to 56.71%, possibly resulting from π-π interactions among C-C bonds with the benzene rings of TC [59]. The O1s spectra (Figure 6f) indicated a decrease in the -OH group percentage from 53.05% to 50.64%, accompanied by a change in binding energy from 531.28 eV up to 531.51 eV, suggesting the participation of hydrogen bonding in the adsorption process [37]. Additionally, as mentioned earlier, the impact of pH on the adsorption capacity of BC-KOH highlighted the role of electrostatic interactions. In general, processes such hydrogen bonding, ion exchange, pore filling, electrostatic attraction, and π-π interactions were the main factors that affected the TC adsorption by BC-KOH (Figure 7).

## 4. Conclusions

This research assessed the effects of acid, alkali, and oxidant treatments on the adsorption capacity of sludge biochar for TC, identifying KOH modification as the most effective enhancement. Porosity, specific surface area, and the presence of oxygen-containing functional groups in the sludge biochar were all significantly enhanced by the KOH treatment. Isothermal adsorption on BC-KOH followed the Langmuir model; however, the TC adsorption kinetics followed the Elovich model. A variety of processes, including π-π interactions, ion exchange, electrostatic attraction, pore filling, and hydrogen bonding, were involved in TC adsorption. The TC adsorption on BC-KOH was negatively affected by ions such as HCO_3_^−^, PO_4_^3−^, Ca^2+^, and Mg^2+^, but very weakly by Cl^−^, NO_3_^−^, and SO_4_^2−^ ions. The greatest adsorption capacity of BC-KOH was 243.32 mg/g at 298 K. There was a lot of real-world potential for BC-KOH because its equilibrium adsorption capacity remained 4.5 times higher than that of unmodified biochar even after three cycles of reuse, with a small 5.94% drop. This work provides new insights into the preparation and application of sludge biochar for treating antibiotic-contaminated wastewater. Nevertheless, further research is required to evaluate the long-term durability and regeneration efficiency of BC-KOH under different environmental conditions as well as its performance in actual wastewater systems with diverse contaminant profiles.

## Figures and Tables

**Table 1:** Pore structure parameters of BC and BC-KOH.

Sample	Specific Surface Area (m^2^/g)	Microporous Surface Area (m^2^/g)	Total Pore Volume (cm^3^/g)	Microporous Volume (cm^3^/g)	Average Pore Width (nm)
BC	43.7194	26.0236	0.082457	0.010699	7.5442
BC-KOH	112.5486	70.3380	0.141910	0.031782	7.5546

**Table 2:** Comparison of removal capacity of TC by various adsorbents.

Biomass	Pyrolysis Condition	Modification Condition	Q_m_ (mg/g)	Reference
Waste chicken feather	450 °C, 1 h	KOH, 800 °C, 1 h	388.33	[44]
Tea waste	700 °C, 2 h	KHCO_3_, 700 °C, 2 h	293.46	[45]
Loblolly pine	300 °C, 15 min	NaOH, 800 °C, 2 h	274.8	[24]
Peanut shell	600 °C, 2 h	KOH, 800 °C	272.21	[46]
Wheat straw	500 °C, 2 h	KOH, 700 °C, 1 h	149.43	[20]
Sawdust	—	ZnCl_2_ + FeCl_3_, 600 °C, 2 h	102.0	[40]
Swine manure	450 °C, 4 h	KMnO_4_, 450 °C, 1 h	95.81	[47]
Suaeda salsa	800 °C, 2 h	FeCl_3_, 25 °C, 3 h	70.17	[16]
Rice husk	500 °C	KOH, 80 °C, 12 h	58.82	[48]
Wheat straw	500 °C, 2 h	KMnO_4_, 700 °C, 1 h	55.59	[20]
Dewatering sludge	600 °C, 2 h	KOH, 105 °C, 12 h	243.32	This Work

Note: The experimental temperatures were all set at 298K. Q_m_ was the maximum adsorption capacity.

**Table 3:** Thermodynamic parameters of the adsorption.

Biochar	T/K	Δ*G*^0^ (kJ/mol)	Δ*H*^0^ (kJ/mol)	Δ*S*^0^ (J/mol·K)
BC	288	3.748	54.985	177.262
	298	2.552		
	308	0.176		
BC-KOH	288	−1.482	58.879	219.748
	298	−2.381		
	308	−3.1889		

## Data Availability

The original contributions presented in the study are included in the article/Supplementary Materials; further inquiries can be directed to the corresponding authors.

## Supplementary Materials

The following Supporting Information can be downloaded at: https://www.mdpi.com/article/10.3390/toxics12100691/s1, Figure S1: Preparation process of modified biochar. The modified biochars were labeled as BC-XX, where XX represented the specific modifier; Figure S2: SEM images of BC (a) and BC-KOH (b); Table S1: Kinetic parameters of TC adsorption by BC and BC-KOH; Table S2: Isotherm parameters of TC adsorption by BC and BC-KOH; Table S3: Pore structure parameters of BC and BC-KOH before and after TC adsorption.

## References

[1] Balakrishnan A., Chinthala M., Polagani R.K., Vo D.-V.N. (2023). Removal of tetracycline from wastewater using g-C_3_N_4_ based photocatalysts: A review. Environ. Res..

[2] Sun Y., Li H., Li G., Gao B., Yue Q., Li X. (2016). Characterization and ciprofloxacin adsorption properties of activated carbons prepared from biomass wastes by H_3_PO_4_ activation. Bioresour. Technol..

[3] Xu L., Zhang H., Xiong P., Zhu Q., Liao C., Jiang G. (2021). Occurrence, fate, and risk assessment of typical tetracycline antibiotics in the aquatic environment: A review. Sci. Total Environ..

[4] Qiao D., Li Z., Duan J., He X. (2020). Adsorption and photocatalytic degradation mechanism of magnetic graphene oxide/ZnO nanocomposites for tetracycline contaminants. Chem. Eng. J..

[5] Fu J., Zhao Y., Yao Q., Addo-Bankas O., Ji B., Yuan Y., Wei T., Esteve-Núñez A. (2022). A review on antibiotics removal: Leveraging the combination of grey and green techniques. Sci Total Environ..

[6] Yu F., Li Y., Han S., Ma J. (2016). Adsorptive removal of antibiotics from aqueous solution using carbon materials. Chemosphere.

[7] Choi Y.-K., Choi T.-R., Gurav R., Bhatia S.K., Park Y.-L., Kim H.J., Kan E., Yang Y.-H. (2020). Adsorption behavior of tetracycline onto *Spirulina* sp. (microalgae)-derived biochars produced at different temperatures. Sci. Total Environ..

[8] Gu C.-H., Pan Y., Wei T.-T., Zhang A.-Y., Si Y., Liu C., Sun Z.-H., Chen J.-J., Yu H.-Q. (2024). Upcycling waste sewage sludge into superior single-atom Fenton-like catalyst for sustainable water purification. Nat. Water.

[9] Tandukar M., Pavlostathis S.G. (2022). Anaerobic co-digestion of municipal sludge with fat-oil-grease (FOG) enhances the destruction of sludge solids. Chemosphere.

[10] Mahmoud M., Ramadan M., Naher S., Pullen K., Baroutaji A., Olabi A.-G. (2020). Recent advances in district energy systems: A review. Therm. Sci. Eng. Prog..

[11] Tang L., Yu J., Pang Y., Zeng G., Deng Y., Wang J., Ren X., Ye S., Peng B., Feng H. (2018). Sustainable efficient adsorbent: Alkali-acid modified magnetic biochar derived from sewage sludge for aqueous organic contaminant removal. Chem. Eng. J..

[12] Li K., Ji F., Liu Y., Tong Z., Zhan X., Hu Z. (2013). Adsorption removal of tetracycline from aqueous solution by anaerobic granular sludge: Equilibrium and kinetic studies. Water Sci. Technol..

[13] Yan L., Liu Y., Zhang Y., Liu S., Wang C., Chen W., Liu C., Chen Z., Zhang Y. (2020). ZnCl_2_ modified biochar derived from aerobic granular sludge for developed microporosity and enhanced adsorption to tetracycline. Bioresour. Technol..

[14] Yang X., Xu G., Yu H., Zhang Z. (2016). Preparation of ferric-activated sludge-based adsorbent from biological sludge for tetracycline removal. Bioresour. Technol..

[15] Gao Y., Yue Q., Gao B., Li A. (2020). Insight into activated carbon from different kinds of chemical activating agents: A review. Sci. Total Environ..

[16] Jiang W., Cai Y., Liu D., Shi Q., Wang Q. (2023). Adsorption properties and mechanism of suaeda biochar and modified materials for tetracycline. Env. Res..

[17] Yu C., Chen X., Li N., Chen J., Yao L., Zhou Y., Lu K., Lai Y., Lai X. (2022). Biomass ash pyrolyzed from municipal sludge and its adsorption performance toward tetracycline: Effect of pyrolysis temperature and KOH activation. Environ. Sci. Pollut. Res..

[18] Liew R.K., Chong M.Y., Osazuwa O.U., Nam W.L., Phang X.Y., Su M.H., Cheng C.K., Chong C.T., Lam S.S. (2018). Production of activated carbon as catalyst support by microwave pyrolysis of palm kernel shell: A comparative study of chemical versus physical activation. Res. Chem. Intermediat..

[19] Shamsuddin M.S., Yusoff N.R.N., Sulaiman M.A. (2016). Synthesis and Characterization of Activated Carbon Produced from Kenaf Core Fiber Using H_3_PO_4_ Activation. Procedia Chem..

[20] Xu J., Zhang Y., Li B., Fan S., Xu H., Guan D.-X. (2022). Improved adsorption properties of tetracycline on KOH/KMnO_4_ modified biochar derived from wheat straw. Chemosphere.

[21] Du L., Ahmad S., Liu L., Wang L., Tang J. (2023). A review of antibiotics and antibiotic resistance genes (ARGs) adsorption by biochar and modified biochar in water. Sci. Total Environ..

[22] Zhang A., Li X., Xing J., Xu G. (2020). Adsorption of potentially toxic elements in water by modified biochar: A review. J. Env. Chem. Eng..

[23] Hoslett J., Ghazal H., Katsou E., Jouhara H. (2021). The removal of tetracycline from water using biochar produced from agricultural discarded material. Sci. Total Environ..

[24] Jang H.M., Yoo S., Choi Y.-K., Park S., Kan E. (2018). Adsorption isotherm, kinetic modeling and mechanism of tetracycline on Pinus taeda-derived activated biochar. Bioresour. Technol..

[25] Zhu X., Liu Y., Qian F., Zhou C., Zhang S., Chen J. (2014). Preparation of magnetic porous carbon from waste hydrochar by simultaneous activation and magnetization for tetracycline removal. Bioresour. Technol..

[26] Reck I.M., Paixão R.M., Bergamasco R., Vieira M.F., Vieira A.M.S. (2018). Removal of tartrazine from aqueous solutions using adsorbents based on activated carbon and *Moringa oleifera* seeds. J. Clean Prod..

[27] Yin Q., Wang R., Zhao Z. (2018). Application of Mg–Al-modified biochar for simultaneous removal of ammonium, nitrate, and phosphate from eutrophic water. J. Clean Prod..

[28] Ma Y., Yang L., Wu L., Li P., Qi X., He L., Cui S., Ding Y., Zhang Z. (2020). Carbon nanotube supported sludge biochar as an efficient adsorbent for low concentrations of sulfamethoxazole removal. Sci. Total Environ..

[29] Zou J., Dai Y., Wang X., Ren Z., Tian C., Pan K., Li S., Abuobeidah M., Fu H. (2013). Structure and adsorption properties of sewage sludge-derived carbon with removal of inorganic impurities and high porosity. Bioresour. Technol..

[30] Wang P., Tang L., Wei X., Zeng G., Zhou Y., Deng Y., Wang J., Xie Z., Fang W. (2017). Synthesis and application of iron and zinc doped biochar for removal of p-nitrophenol in wastewater and assessment of the influence of co-existed Pb(II). Appl. Surf. Sci..

[31] Song X., Hu Y., Zheng M., Wei C. (2016). Solvent-free in situ synthesis of g-C_3_N_4_/{001}TiO_2_ composite with enhanced UV- and visible-light photocatalytic activity for NO oxidation. Appl. Catal. B-Environ..

[32] Ge Q., Li P., Liu M., Xiao G.-m., Xiao Z.-q., Mao J.-w., Gai X.-k. (2023). Removal of methylene blue by porous biochar obtained by KOH activation from bamboo biochar. Bioresour. Bioprocess..

[33] Luo J., Li X., Ge C., Müller K., Yu H., Huang P., Li J., Tsang D.C.W., Bolan N.S., Rinklebe J. (2018). Sorption of norfloxacin, sulfamerazine and oxytetracycline by KOH-modified biochar under single and ternary systems. Bioresour. Technol..

[34] Liu H., Xu G., Li G. (2020). The characteristics of pharmaceutical sludge-derived biochar and its application for the adsorption of tetracycline. Sci. Total Environ..

[35] Zhang C., Lai C., Zeng G., Huang D., Yang C., Wang Y., Zhou Y., Cheng M. (2016). Efficacy of carbonaceous nanocomposites for sorbing ionizable antibiotic sulfamethazine from aqueous solution. Water Res..

[36] Ouyang D., Chen Y., Yan J., Qian L., Han L., Chen M. (2019). Activation mechanism of peroxymonosulfate by biochar for catalytic degradation of 1,4-dioxane: Important role of biochar defect structures. Chem. Eng. J..

[37] Su X., Wang X., Ge Z., Bao Z., Lin L., Chen Y., Dai W., Sun Y., Yuan H., Yang W. (2024). KOH-activated biochar and chitosan composites for efficient adsorption of industrial dye pollutants. Chem. Eng. J..

[38] Xiong S., Gong D., Deng Y., Tang R., Li L., Zhou Z., Zheng J., Yang L., Su L. (2021). Facile one-pot magnetic modification of Enteromorpha prolifera derived biochar: Increased pore accessibility and Fe-loading enhances the removal of butachlor. Bioresour. Technol..

[39] Ma J., Zhou B., Zhang H., Zhang W. (2020). Fe/S modified sludge-based biochar for tetracycline removal from water. Powder Technol..

[40] Zhou Y., Liu X., Xiang Y., Wang P., Zhang J., Zhang F., Wei J., Luo L., Lei M., Tang L. (2017). Modification of biochar derived from sawdust and its application in removal of tetracycline and copper from aqueous solution: Adsorption mechanism and modelling. Bioresour. Technol..

[41] Chang J., Shen Z., Hu X., Schulman E., Cui C., Guo Q., Tian H. (2020). Adsorption of Tetracycline by Shrimp Shell Waste from Aqueous Solutions: Adsorption Isotherm, Kinetics Modeling, and Mechanism. ACS Omega.

[42] Ramanayaka S., Tsang D.C.W., Hou D., Ok Y.S., Vithanage M. (2020). Green synthesis of graphitic nanobiochar for the removal of emerging contaminants in aqueous media. Sci. Total Environ..

[43] Selahle S.K., Waleng N.J., Mpupa A., Nomngongo P.N. (2020). Magnetic Solid Phase Extraction Based on Nanostructured Magnetic Porous Porphyrin Organic Polymer for Simultaneous Extraction and Preconcentration of Neonicotinoid Insecticides from Surface Water. Front. Chem..

[44] Li H., Hu J., Meng Y., Su J., Wang X. (2017). An investigation into the rapid removal of tetracycline using multilayered graphene-phase biochar derived from waste chicken feather. Sci Total Environ..

[45] Li B., Huang Y., Wang Z., Li J., Liu Z., Fan S. (2021). Enhanced adsorption capacity of tetracycline on tea waste biochar with KHCO_3_ activation from aqueous solution. Environ. Sci. Pollut. Res..

[46] Zhang Y., Xu J., Li B., Xie Z., Li X., Tang J., Fan S. (2023). Enhanced adsorption performance of tetracycline in aqueous solutions by KOH-modified peanut shell-derived biochar. Biomass Convers. Biorefinery.

[47] Fu Z., Chen Y., Lu Y., Wang Y., Chen J., Zhao Y., Yang M., Tian X. (2022). KMnO_4_ modified biochar derived from swine manure for tetracycline removal. Water Pract. Technol..

[48] Liu P., Liu W.-J., Jiang H., Chen J.-J., Li W.-W., Yu H.-Q. (2012). Modification of bio-char derived from fast pyrolysis of biomass and its application in removal of tetracycline from aqueous solution. Bioresour. Technol..

[49] Zhang P., Li Y., Cao Y., Han L. (2019). Characteristics of tetracycline adsorption by cow manure biochar prepared at different pyrolysis temperatures. Bioresour. Technol..

[50] Nie Y., Zhao C., Zhou Z., Kong Y., Ma J. (2023). Hydrochloric acid-modified fungi-microalgae biochar for adsorption of tetracycline hydrochloride: Performance and mechanism. Bioresour. Technol..

[51] Zeng Z., Ye S., Wu H., Xiao R., Zeng G., Liang J., Zhang C., Yu J., Fang Y., Song B. (2019). Research on the sustainable efficacy of g-MoS_2_ decorated biochar nanocomposites for removing tetracycline hydrochloride from antibiotic-polluted aqueous solution. Sci. Total Environ..

[52] Dai J., Meng X., Zhang Y., Huang Y. (2020). Effects of modification and magnetization of rice straw derived biochar on adsorption of tetracycline from water. Bioresour. Technol..

[53] Chen Z., Jing Y., Wang Y., Meng X., Zhang C., Chen Z., Zhou J., Qiu R., Zhang X. (2020). Enhanced removal of aqueous Cd(II) by a biochar derived from salt-sealing pyrolysis coupled with NaOH treatment. Appl. Surf. Sci..

[54] Zhao J., Liang G., Zhang X., Cai X., Li R., Xie X., Wang Z. (2019). Coating magnetic biochar with humic acid for high efficient removal of fluoroquinolone antibiotics in water. Sci. Total Environ..

[55] Afzal M.Z., Yue R., Sun X.-F., Song C., Wang S.-G. (2019). Enhanced removal of ciprofloxacin using humic acid modified hydrogel beads. J. Colloid. Interf. Sci..

[56] Wang K., Yao R., Zhang D., Peng N., Zhao P., Zhong Y., Zhou H., Huang J., Liu C. (2023). Tetracycline Adsorption Performance and Mechanism Using Calcium Hydroxide-Modified Biochars. Toxics.

[57] Liang J., Fang Y., Luo Y., Zeng G., Deng J., Tan X., Tang N., Li X., He X., Feng C. (2019). Magnetic nanoferromanganese oxides modified biochar derived from pine sawdust for adsorption of tetracycline hydrochloride. Environ. Sci. Pollut. Res..

[58] Wang Q., Yue Y., Liu W., Liu Q., Song Y., Ge C., Ma H. (2023). Removal Performance of KOH-Modified Biochar from Tropical Biomass on Tetracycline and Cr(VI). Materials.

[59] Chen H., Gao Y., El-Naggar A., Niazi N.K., Sun C., Shaheen S.M., Hou D., Yang X., Tang Z., Liu Z. (2022). Enhanced sorption of trivalent antimony by chitosan-loaded biochar in aqueous solutions: Characterization, performance and mechanisms. J. Hazard. Mater..

[60] Zhou L., Zhang G., Zeng Y., Bao X., Liu B., Cheng L. (2024). Endogenous iron-enriched biochar derived from steel mill wastewater sludge for tetracycline removal: Heavy metals stabilization, adsorption performance and mechanism. Chemosphere.

