# Supplementary material for: Enhanced Tetracycline Adsorption Using KOH-Modified Biochar Derived from Waste Activated Sludge in Aqueous Solutions
Source: Toxics. 2024 Sep 25;12(10):691. doi: 10.3390/toxics12100691 (PMC11511317; doi:10.3390/toxics12100691)
Supplement: Supplementary file 1 [file toxics-12-00691-s001.zip › toxics-3212370-supplementary.pdf]

Supplementary information

Calculation methods

The adsorption capacity of TC was calculated according to (Eq.S1).

$$Q_e = \frac{(C_o - C_e)V}{M} \tag{S1}$$

where C_0 and C_e are the concentrations of TC (mg/L) before and after adsorption; V is the initial solution volume (L); and M is the weight of the sludge biochar (g).

The pseudo-first-order kinetics model (Eq.S2), pseudo-second-order kinetics model (Eq.S3), Elovich kinetics model (Eq.S4), intra-particle diffusion model (Eq.S5), and liquid film diffusion model (Eq.S6) were used to fit the adsorption curve.

$$ln(Q_e - Q_t) = lnQ_e - \frac{k_1}{2.303}t \tag{S2}$$

$$\frac{t}{Q_t} = \frac{1}{k_2 {Q_e}^2} + \frac{t}{q_e} \tag{S3}$$

$$Q_e = \frac{1}{\beta} ln(\alpha\beta) + \frac{1}{\beta} lnt \tag{S4}$$

$$Q_t = k_p t^{0.5} + C \tag{S5}$$

$$ln\left(1 - \frac{Q_t}{Q_e}\right) = -k_{fd}t \tag{S6}$$

Where k_1, k_2, k_p, and K_{fd} are the pseudo-first-order constant, the equilibrium constant of pseudo-second-order, intra-particle diffusion constant and adsorption rate constant; Q_e is adsorption capacity at equilibrium time, mg/g; t is adsorption time, min; Q_t is adsorption capacity at time t, mg/g; α and β are the rate constants of diverse kinetics; C is to describe the boundary layer characteristic.

The Langmuir adsorption isotherm model (Eq.S7), Freundlich adsorption isotherm model (Eq.S8), and Temkin model (Eq.S9) were used to fit the adsorption isotherm data.

$$Q_e = \frac{k_L Q_m C_e}{1+k_L C_e} \qquad \text{（S7）}$$

$$Q_e = k_F C_e^{\frac{1}{n}} \qquad \text{（S8）}$$

$$Q_e = \frac{RT}{b} ln\ (K_T C_e) \qquad \text{（S9）}$$

where Q_m is the maximum amount of TC, mg/g, K_L is the Langmuir constant related to the energy of adsorption, L/mg; C_e is the equilibrium concentration of TC, mg/L; K_F is the Freundlich constant denoting adsorption capacity, mg/g $(\mathrm{L/mg})^{1/n}$; n is the empirical constant, indicating of adsorption intensity; R is the ideal gas constant, 8.314 J/(mol·K); b is Temkin constant; K_T is equilibrium bond constant related to the maximum energy of bond, L/g; T is temperature in terms of Kelvin, K.

The calculation formulas of Gibbs free energy (ΔG^0), enthalpy change (ΔH^0) and entropy change (ΔS^0) were shown in Eq.S10-S13.

$$\Delta G^0 = -\mathrm{R}T\ln K_\mathrm{d} \qquad \text{(S10)}$$

$$\Delta G^0 = \Delta H^0 - T\Delta S^0 \qquad \text{(S11)}$$

$$\ln K_\mathrm{d} = \frac{\Delta S^0}{R} \quad \frac{\Delta H^0}{RT} \qquad \text{(S12)}$$

$$K_\mathrm{d} = \frac{Q_\mathrm{e}}{C_\mathrm{e}} \qquad \text{(S13)}$$

Where the ΔG^0 is the standard Gibbs free energy, kJ/mol; ΔH^0 is the enthalpy change, kJ/mol; ΔS^0 is the entropy change, J/(mol·K); K_d is adsorption equilibrium constant, L/mol.

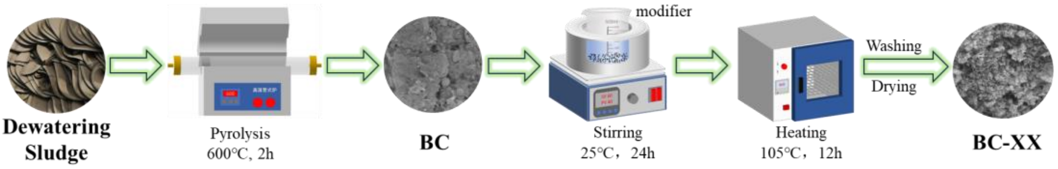

Fig S1. Preparation process of modified biochar. The modified biochars were labeled as BC-XX, where XX represented the specific modifier.

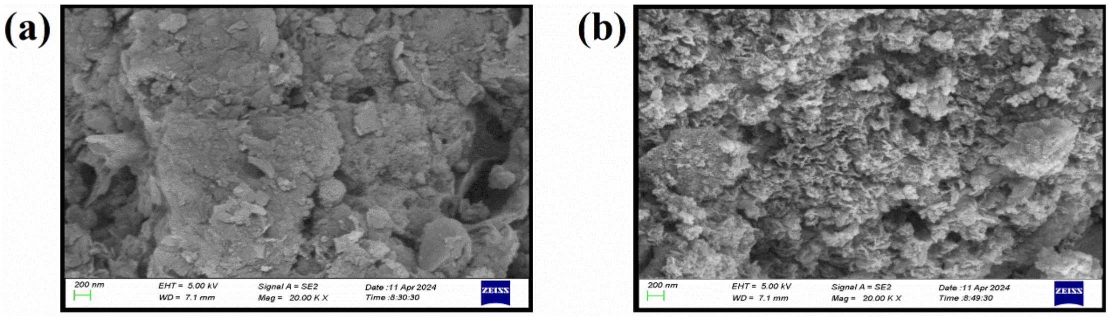

Fig S2. SEM images of BC (a) and BC-KOH (b).

Table S1 Kinetic parameters of TC adsorption by BC and BC-KOH.

**Fitting model**	**Parameter**	**BC**	**BC-KOH**
Pseudo-first-order model	$Q_{t,cal}$ (mg/g)	11.50	92.99
	k_L (mg/(g·min))	0.0033	0.0023
	R^2	0.9289	0.9521
Pseudo-second-order model	$Q_{t,cal}$ (mg/g)	32.53	145.35
	K_2 (mg/(g·min))	0.0011	0.0001
	R^2	0.9996	0.9875
Elovich model	α (mg/(g·min))	255.04	258.37
	β (g/mg)	0.2689	0.0454
	R^2	0.9661	0.9876
Intraparticle model	K_{id1} $(mg/(g\ min^{1/2})$	2.2898	13.0996
	C_1 (mg/g)	6.8561	-15.2443
	R_1^2	0.9997	0.9974
	K_{id2} $(mg/(g\ min^{1/2})$	0.8164	3.9783
	C_2 (mg/g)	16.271	37.2969
	R_2^2	0.9765	0.9536
	K_{id3} $(mg/(g\ min^{1/2})$	0.1170	2.17076
	C_3 (mg/g)	27.6893	60.1650
	R_3^2	0.99935	0.9996
Liquid membrane diffusion models	K_{fd}	0.0033	0.0023
	R_2	0.9289	0.9521

Table S2 Isotherm parameters of TC adsorption by BC and BC-KOH.

Biochar	T	Langmuir			Freundlich			Temkin			
	K	Q_m (mg/g)	K_L (L/mg)	R^2	n	K_F (mg/g)	R^2	K_T	b	RT/b	R^2
BC	288	18.609	0.094	0.935	0.297	0.673	0.997	1.243	0.677	3.537	0.988
	298	31.064	0.118	0.888	0.293	0.915	0.993	1.785	0.418	5.728	0.965
	308	62.605	0.067	0.977	0.441	0.952	0.996	0.833	0.190	12.602	0.974
BC-KOH	288	121.049	0.211	0.990	0.383	1.441	0.943	3.732	0.113	21.190	0.989
	298	243.320	0.067	0.987	0.478	1.483	0.965	2.336	0.067	35.738	0.912
	308	303.901	0.045	0.989	0.543	1.419	0.984	1.343	0.005	45.178	0.910

Table S3 Pore structure parameters of BC and BC-KOH before and after TC adsorption.

Parameter	BET surface area （m^2/g）	Microporous surface area （m^2/g）	Total pore volume （cm^3/g）	Microporous volume （cm^3/g）
Before adsorption	112.5486	70.3380	0.141910	0.031782
After adsorption	85.5985	70.9541	0.096120	0.030612
